# Microglial Melatonin Receptor 1 Degrades Pathological Alpha‐Synuclein Through Activating LC3‐Associated Phagocytosis In Vitro

**DOI:** 10.1111/cns.70088

**Published:** 2024-10-23

**Authors:** Xiao‐Yu Yao, Bing‐Er Cao, Jun‐Yi Liu, Qian‐Kun Lv, Jia‐Rui Zhang, Xiao‐Yu Cheng, Cheng‐Jie Mao, Quan‐Hong Ma, Fen Wang, Chun‐Feng Liu

**Affiliations:** ^1^ Department of Neurology and Clinical Research Center of Neurological Disease The Second Affiliated Hospital of Soochow University Suzhou China; ^2^ Jiangsu Key Laboratory of Neuropsychiatric Diseases and Institute of Neuroscience Soochow University Suzhou China; ^3^ Department of Neurology The Fourth Affiliated Hospital of Soochow University Suzhou China; ^4^ Department of Neurology Xiongan Xuanwu Hospital Xiongan China

**Keywords:** LC3‐associated phagocytosis, melatonin receptor 1, microglia, Parkinson's disease, α‐synuclein

## Abstract

**Aims:**

Parkinson's disease (PD) is characterized by the formation of Lewy bodies (LBs), primarily constituted of α‐synuclein (α‐Syn). Microglial cells exhibit specific reactivity toward misfolded proteins such as α‐Syn. However, the exact clearance mechanism and related molecular targets remain elusive.

**Methods:**

BV2 cells, primary microglia from wild‐type and MT1 knockout mice, and primary cortical neurons were utilized as experimental models. The study investigated relevant mechanisms by modulating microglial MT1 expression through small RNA interference (RNAi) and lentiviral overexpression techniques. Furthermore, pathological aggregation of α‐Syn was induced using pre‐formed fibrils (PFF) α‐Syn. Co‐immunoprecipitation, immunofluorescence, Western blot (WB), and quantitative real‐time PCR were used to elucidate the mechanisms of molecular regulation.

**Results:**

In this study, we elucidated the regulatory role of the melatonin receptor 1 (MT1) in the microglial phagocytic process. Following MT1 knockout, the ability of microglial cells to engulf latex beads and zymosan particles decreased, subsequently affecting the phagocytic degradation of fibrillar α‐Syn by microglial cells. Furthermore, the loss of MT1 receptors in microglial cells exacerbates the aggregation of α‐Syn in neurons induced by pre‐formed fibrils (PFF) α‐Syn. Mechanistically, MT1 influences the phagocytic function of microglial cells by regulating the Rubicon‐dependent LC3‐associated phagocytosis (LAP) pathway.

**Conclusion:**

Taken together, the results suggest the neuroprotective function of microglial cells in clearing α‐Syn through MT1‐mediated LAP, highlighting the potential key role of MT1 in pathogenic mechanisms associated with α‐Syn.

## Graphical abstract

A model illustrating the role of microglial MT1 in α‐Synuclein clearance: Microglial MT1 can recognize and internalize the pathological α‐Syn released by dopaminergic neurons, which is related to the activation of LC3‐associated phagocytosis. However, when MT1 functionality is compromised, the LAP process fails to proceed effectively, thereby impeding the degradation and clearance of α‐Syn.

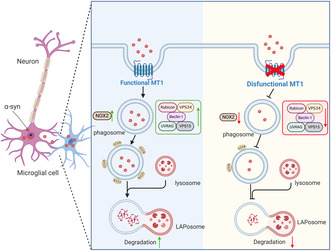

**Abbreviations**
AD: Alzheimer's disease
Aβ: amyloid‐β
Co‐IP: co‐immunoprecipitation
Iba1: ionized calcium‐binding adapter molecule 1
IF: immunofluorescence
LAP: LC3‐associated phagocytosis
LC3: microtubule‐associated protein 1 light chain 3
LV: lentivirus
MAP 2: microtubule‐associated protein 2
MG: primary microglia
MT1: melatonin receptor 1
MT1‐KO: MT1 knockout
PBS: phosphate buffer saline
PD: Parkinson's disease
PFA: paraformaldehyde
PFF: pre‐formed fibrils
ps129: α‐Syn phosphorylated on serine 129
RT‐qPCR: reverse transcription quantitative real‐time polymerase chain reaction
SN: substantia nigra
WB: Western blotting
WT: wild type
α‐Syn: alpha‐synuclein

## 1 Introduction

Parkinson's disease (PD) is the second most common neurodegenerative disease affecting the health of the elderly. It is characterized by the progressive loss of dopaminergic neurons in the substantia nigra (SN) and the formation of Lewy bodies (LBs), where fibrillar alpha‐synuclein (α‐Syn) aggregates serve as a predominant protein component within the surviving neurons [1, 2, 3]. In recent years, a plethora of clinical studies and evidence from animal models have indicated that changes in α‐Syn occur early in the onset of PD [4, 5, 6]. The misfolded pathological α‐Syn can spread between neighboring neurons in anatomically connected brain regions, acting as “pathological seeds” and triggering a cascade of detrimental reactions, thereby aggravating the onset and progression of PD [7, 8, 9]. Therefore, the clearance of α‐Syn aggregates is crucial for both preventing and treating PD; underscoring the pursuit of more effective clearance methods has also become a crucial unresolved scientific challenge.

In the brain, glial cells, in addition to neurons, play a pivotal role in maintaining homeostasis and supporting various functions. Recent studies indicate that the dysfunction of glial cells exacerbates the onset and progression of neurodegeneration diseases, including PD [10, 11]. Microglia, a dynamic immune cell population in the central nervous system (CNS) [12, 13, 14], actively engage in chemotaxis, phagocytosis, and the secretion of inflammatory cytokines. Concurrently, they perpetually surveil the brain, serving as “guardians” within the parenchyma, detecting damage and safeguarding brain tissue through local phagocytic activities [15, 16]. The efficient clearance of extracellular debris, pathogens, and toxic protein aggregates by microglial cells is pivotal, relying significantly on their phagocytic function [17]. Some researchers have revealed that in the mouse model of PD, microglial cells influence the intercellular transfer of α‐Syn [18]. In addition, microglial cells can uptake pathological proteins such as α‐Syn released by neurons through various pathways, including selective autophagy, the TLR2/TLR4‐NF‐κB signaling pathway, tunneling nanotubes, thereby preventing the formation of LBs [19, 20, 21, 22, 23, 24, 25, 26]. In recent years, a novel engulfment pathway, namely LC3‐associated phagocytosis (LAP), has emerged as a subject of interest within the scientific community. LAP, characterized as a non‐canonical autophagy pathway, assumes a pivotal role in eliminating extracellular particles, encompassing apoptotic cells, pathogens, and aberrantly aggregated proteins [27, 28]. Researchers have observed that LAP exhibits the capability to facilitate the clearance of β‐amyloid protein (Aβ) and Tau protein in the Alzheimer's disease (AD) mouse model, thereby alleviating neurodegenerative lesions [25, 29]. Furthermore, LAP has been demonstrated to promote the degradation of axon debris by glial cells in *Drosophila* models after injury [30]. In mice, microglial debris is degraded via Rubicon‐dependent LAP [31]. Nevertheless, the role of LAP in PD remains uncertain. Additionally, whether there are molecules or receptors influence LAP and consequently impact the clearance of pathological α‐Syn is a question worthy of further investigation.

Melatonin receptor 1 (MT1) serves as one of the affinity receptors for melatonin, functioning as a G protein‐coupled receptor. Its involvement extends across a spectrum of physiological and pathological processes, encompassing circadian rhythm, sleep, emotions, learning and memory, neuroprotection, and cancer, among others [32, 33, 34, 35]. Notably, studies indicate a tendency for a decrease in MT1 expression with age [36]. In neurodegeneration diseases, melatonin has been demonstrated to prevent mitochondrial dysfunction, exerting neuroprotective effects [37, 38, 39, 40]. Furthermore, in individuals with PD, a reduction in MT1 expression has been observed in the SN and amygdala, highlighting the potential significance of MT1 in aging‐related diseases [41]. Our prior research has unveiled diminished MT1 expression levels in the SN of PD mouse models, and the activation of MT1 was found to inhibit lipopolysaccharide‐induced microglial activation, influencing their metabolic reprogramming [42]. This discovery underscores a strong correlation between microglial MT1 and the pathological progression of PD. Additionally, MT1 has been identified as playing a crucial role in regulating the production of the pathogenic protein Aβ associated with AD. MT1 mutations have been evidenced to expedite Aβ deposition in neurons in AD animal models [43]. However, the impact of MT1 on the pathogenic protein α‐Syn in PD has yet to be fully elucidated. The ligand of MT1, melatonin, has been shown to reduce α‐Syn aggregation in PD animal models and actively participates in modulating the phagocytic process of microglial cells [44, 45, 46]. Consequently, MT1 is likely to regulate the phagocytic activity of microglia, thereby alleviating the pathological aggregation of α‐Syn and reversing the progression of PD.

In this study, we delved into the relationship between the phagocytic function of microglial cells and the clearance of pathological α‐Syn. Our results sheds light on the regulatory role of MT1 in the phagocytic function of microglial cells, intricately connected to LAP. Significantly, the deficiency of MT1 was observed to heighten the aggregation of α‐Syn and impair the clearance of pathological α‐Syn aggregates within neurons by microglial cells. Thus, this study provides valuable insights for maintaining the dynamic balance of the CNS and it opens avenues for potential therapeutic strategies in addressing PD.

## 2 Materials and Methods

### 2.1 Ethics Approval Statement

This study does not contain any human data. All animal experiment protocols have been approved by the Institutional Animal Care and Use Committee of Soochow University (Suzhou, China).

### 2.2 Animals

*Mtnr1a* knock‐out (MT1‐KO) mice were procured from GemPharmatech Co., Ltd. Laboratory (Nanjing, China). The model was generated using CRISPR/Cas9 technology to disrupt the exon 2 region of the Mtnr1a‐201 (ENSMUST00000067984.8) transcript. The concise procedure is outlined as follows: sgRNA was transcribed in vitro, and Cas9 along with sgRNA were microinjected into the fertilized eggs of C57BL/6JGpt mice. The fertilized eggs were subsequently transplanted to obtain positive F0 mice, whose genetic alteration was verified through PCR and sequencing. A stable F1 generation mouse model was established by mating positive F0 generation mice with C57BL/6JGpt mice. The animals were housed under controlled conditions, with temperatures maintained between 20°C and 26°C, relative humidity set at 50%–60%, and a 12‐h light/dark cycle in individually ventilated cages. Throughout the study, ad libitum access to food and water was provided to the animals.

### 2.3 Cell Culture

#### 2.3.1 BV2 Cell Line

Murine BV2 microglia cells were graciously supplied by Prof. Guanghui Wang from the Department of Pharmacology at Soochow University, China. BV2 cells were cultured in Dulbecco's modified Eagle's medium (DMEM, 11965092, Gibco, USA), supplemented with 10% heat‐inactivated fetal bovine serum (FBS, 10099141C, Gibco, USA) and 1% penicillin/streptomycin (15140122, Gibco, USA). The cells were nurtured in a humidified incubator at 37°C with 5% CO_2_.

#### 2.3.2 Primary Microglial Cells

The primary microglial and astrocyte mixed cultures were obtained from the brains of neonatal C57BL/6JGpt wild‐type (Mtnr1a^+/+^) or Mtnr1a knock‐out (Mtnr1a^−/−^) pups aged P1, whose isolation procedure was carried out by a previously published article [42]. In brief, the meninges and blood vessels of brain cortices were carefully dissociated in ice‐cold phosphate‐buffered saline (PBS). The cortices were then minced into small pieces and digested with 0.25% Trypsin (25200072, Gibco, USA) for 15 min at 37°C. Following tissue digestion, the suspension was filtered through a 70 μm cell strainer, and the cell suspensions were centrifuged at 1000 rpm for 10 min. The resulting mixed glial cell pellets were resuspended in DMEM containing 10% heat‐inactivated FBS and 1% penicillin/streptomycin. Subsequently, cells isolated from cortices tissue were plated on poly‐d‐lysine‐coated T‐75 flasks (Corning, Tewksbury, MA, USA) and cultured at 37°C, 5% CO_2_. The culture mediums were replaced after 24 hours the first time and then every 3 days. After 10–12 days, primary microglial cells were separated from the mixed glial cells by shaking at 220 rpm for 1.5 h at 37°C. Isolated primary microglial cells were then cultured in well plates at the density of 2 × 10^5^ cells per well (24‐well plate) or 4 × 10^5^ cells per well (12‐well plate) for further experiments.

#### 2.3.3 Primary Neuron

Primary neuronal cultures were generated from the brains of neonatal C57BL/6JGpt mice aged P1 to P3. The isolation procedure closely resembled that of primary microglial cells. Cortical hippocampal neurons were dissociated and subsequently seeded onto poly‐d‐lysine‐coated coverslips or dishes at cell densities of 2 × 10^5^ cells/well (24‐well plate) or 7 × 10^5^ cells/well (six‐well plate), respectively. The preponderance of experiments was carried out at 15 days in vitro (DIV).

### 2.4 Purification of Human α‐Syn PFFs

Recombinant human α‐Syn PFF was purified using a previously established protocol and stored at −80°C until use [47]. In summary, the plasmid pET21a, containing human α‐Syn cDNA (addgene plasmid 51486, Michael J. Fox Foundation, USA), was transformed into BL21 (DE3) Escherichia coli (TransGen Biotech, China). Alpha‐synuclein protein expression was induced with 0.25 mM isopropyl‐1‐thio‐d‐galactopyranoside (IPTG) for 4 h at 37°C. The cell pellet was lysed, and α‐Syn monomers were subsequently filtered with a 0.22‐μm membrane (SLGP033RB, Millipore, MA, USA) and purified by HiTrap Q FF anion exchange chromatography column (GE Healthcare, PA, USA). To obtain α‐Syn PFF, the purified α‐Syn monomers were diluted in PBS (5 mg/mL) and agitated for 7 days (1000 rpm, 37°C) to facilitate the formation of mature fibrils. Subsequently, the aggregates were collected by centrifugation, resuspended in endotoxin‐free PBS, and sonicated for 1 min into α‐Syn PFF (1 s on, 1 s off, 20% amplitude) using a sonicator. The concentration of α‐Syn was determined by a bicinchoninic acid (BCA) protein assay kit (23225; Thermo Scientific, MA, USA). To confirm aggregate formation, α‐Syn PFF before and after sonication were applied to carbon‐coated copper grids and left to stand for 2 min, followed by staining with 2% phosphotungstic acid (G1870, Solarbio, China) for 2 min. Micrographs were taken with a transmission electron microscope (TECNAI G2 F20, FEI, USA) at 200 kV and 34,000 × magnification.

### 2.5 Neuron‐Microglial Co‐Culture

The experimental method was adapted from the literature [48] with some modifications. Initially, primary neurons were seeded at a density of 2 × 10^5^ cells (24‐well plate) or 1 × 10^6^ cells (six‐well plate). On the 6th day of neuron culture, α‐Syn PFF was introduced at a concentration of 2 μg/mL.

For the preparation of primary microglial cells from both wild‐type (WT) and MT1‐KO mice, we followed the outlined procedure. After centrifugation at 1000 rpm for 5 min, microglia were resuspended in neurobasal complete media and then plated onto 15 days in vitro (DIV) primary neurons. The seeding ratio of primary microglial cells to neurons was maintained at 1:3, with 7 × 10^4^ cells seeded in 24‐well plates and 3.5 × 10^5^ cells in six‐well plates. Co‐cultures were either fixed or collected for subsequent experiments 3 days after microglia plating.

We established a primary neuron–microglia co‐culture model, inducing pathological α‐Syn aggregation in neurons by introducing α‐Syn PFF (2 μg/mL) on the 5th day of neuron culture. After 7 days of α‐Syn PFF treatment, WT and MT1‐KO primary microglial cells were introduced for a 3‐day co‐culture (Figure 5A).

### 2.6 RNA Interference

RNAiMAX (13778100, Invitrogen, CA, USA) is used for the transfection of BV2 cells or primary microglial cells with RNA oligonucleotides. The cells were incubated in a mixture of RNAiMAX, Opti‐MEM, and RNA oligonucleotides for 5 min at room temperature. After 24 hours, the media were replaced with fresh media. Another 24 hours later, the cells were collected for subsequent experiments. The oligonucleotides that target mouse *Mtnr1a* were purchased from Sangon Biotech, whose sequences were as follows: negative control: sense: UUCUCCGAACGUGUCACGU, anti‐sense: ACGUGACACGUUCGGAGAA; si‐*Mtnr1a*: sense: GCUGCCUCAACGCAAUUAUAU, anti‐sense: AUAUAAUUGCGUUGAGGCAGC.

### 2.7 Lentiviral Transduction and Stable MT1‐Expressing Strain Construction

The lentivirus for overexpressing MT1(LV‐MT1) and the control lentivirus (LV‐GFP) were obtained commercially from GenePharma Corporation (Shanghai, China). To overexpress MT1, BV2 cells were infected with Ubi‐MCS‐3FLAG‐CBh‐gcGFP‐IRES‐puromycin lentiviral particles (Gene ID: 17773, Genechem Co., Ltd., China) for 24 h, BV2 cells that were not effectively infected were killed by adding 2.5 μg/mL of puromycin after 120 h to obtain a stable MT1‐expressing strain. The expression of the MT1 was confirmed by RT‐qPCR and western blot. The primary microglial cells were transfected with lentivirus to overexpress MT1 using a similar method.

### 2.8 Phagocytic Activity Assay

#### 2.8.1 Fluorescence Latex Bead Phagocytosis

The latex beads phagocytosis assay was modified from methods described by other researchers [49]. In brief, BV2 cells and primary microglial cells were seeded in 24‐well plates at a density of 2 × 10^5^ cells for 24 h. Subsequently, fluorescence latex beads (L2778, Sigma, St. Louis, MO, USA) were incubated at 37°C in a solution comprising 50% FBS and PBS for 1 h. The latex beads were subsequently added at a concentration of 1:1000 for BV2 cells and 1:2000 for primary microglial cells, followed by incubation at 37°C for 2 h (BV2) or 3 h (primary microglial cells). In the end, the culture medium and non‐phagocytosed latex beads were gently removed by washing with pre‐chilled 1 × PBS on a shaker, performing five washes, each lasting 10 min. Cells intended for immunofluorescence examination were fixed at room temperature using 4% paraformaldehyde (PFA) and then photographed under a confocal microscope (Zeiss, LSM 900, Carl Zeiss, Jena, Germany).

#### 2.8.2 Zymosan Phagocytosis

The procedure is similar to the latex beads phagocytosis method mentioned earlier, which was modified from methods described by other researchers [50]. Briefly, cells were incubated at 37°C with 30 μg/mL of the fluorescent zymosan particle (IAK0111, Sigma, California, USA). BV2 cells and primary microglial cells were incubated for 3 and 1.5 h, respectively. Following the incubation, cells underwent five washes with cold PBS, with each wash lasting 10 min, to remove unbound particles. The cells intended for immunofluorescence were fixed with 4% PFA at room temperature, sealed with a mounting medium containing DAPI, and observed under a Zeiss microscope, with images captured.

### 2.9 Western Blotting

After treatment, cells were harvested and lysed at 4°C in an ice‐cold Cell Lysis Buffer (P0013B, Beyotime, Shanghai, China) containing a protease inhibitor cocktail (HY‐K0010, MCE, Shanghai, China) for 30 min and then centrifuged at 13,200 rpm for 15 min at 4°C. A BCA protein assay kit was used to detect the protein concentration of the supernatants. For Triton X‐100‐soluble and ‐insoluble α‐Syn detection, we lysed the tissues in 1 × TBS with 1% Triton X‐100 and protease and phosphatase inhibitors, centrifuged at 20,000 rpm for 1 h at 4°C, and the supernatants were collected as Triton X‐100‐soluble samples. The pellets were resuspended in 1 × TBS with 2% SDS containing protease and phosphatase inhibitors as Triton X‐100‐insoluble samples. The samples were prepared with 5 × loading buffer (FD002; Fude, Hangzhou, China) for 5 min at 95°C. Subsequently, 30 μg of protein in each lysate sample was subjected to SDS‐PAGE (Bio‐Rad) at 110 V and then transferred to a polyvinylidene fluoride (PVDF) membrane (ISEQ00010, Millipore, MA, USA). The PVDF membrane was blocked with 5% nonfat dry milk, and then incubated overnight at 4°C with the following primary antibodies: α‐Syn (1:1000; ab1903, Abcam, Cambridge, UK), MT1 (1:1000; A13030, Abclonal, Wuhan, China), MT2 (1:500; abs120311, Absin, Shanghai, China), Rubicon (1:1000; D9F7, CST, MA, USA), PI3KC‐III (1:1000; D9A5, CST, MA, USA), LC3B (1:1000; NB100‐2220125066, NOVUS, Colorado, USA), UVRAG (1:1000; D2Q1Z, CST, MA, USA), NOX2 (1:1000; AF2290, Beyotime, Shanghai, China), Beclin‐1 (1:1000; D40C5, CST, MA, USA), PIK3R4 (1:1000; 14580, CST, MA, USA), ATG5 (1:1000; 12994S, CST, MA, USA), β‐actin (1:5000; A3854, Sigma, St. Louis, MO, USA), GAPDH (1:5000; AC002, Abclonal, Wuhan, China). The membrane was washed three times in TBST buffer and then incubated with an HRP‐linked anti‐mouse IgG secondary antibody (1:10,000; 115‐001‐003, Jackson Immuno Research Laboratories, PA, USA) or HRP‐linked anti‐rabbit IgG secondary antibody (1:10,000; 111‐001‐003, Jackson Immuno Research Laboratories, PA, USA) for 1 h at room temperature. The protein bands were visualized by ECL detection reagents (P10300; NCM Biotech, Suzhou, China). GAPDH, or β‐actin, was used as a housekeeping control for whole‐cell lysates.

### 2.10 RNA Extraction, Reverse Transcription‐PCR, and Real‐Time Quantitative RT‐PCR

After different treatments of BV2 cells, the total RNA was obtained using TRIzol Reagent (15596018; Invitrogen, CA, USA), which was then reverse transcribed by the cDNA synthesis kit (K1622; Thermo Scientific, MA, USA). The real‐time quantitative PCR was performed using SYBR Green PCR Master Mix (A25778; ABI, CA, USA), and the samples were measured using a 7500 Real Time PCR System (ABI, CA, USA). The sequences of PCR primers (Genewiz, Suzhou, China) were used as shown in Table 1. The data were normalized to *18s* RNA, and the fold change was calculated via the $2^{-∆∆C_{t}}$ method. Based on the untreated group, the relative concentration of mRNA was expressed in arbitrary units, and its assigned value was 1.

**TABLE 1:** PCR primer sequences used.

Gene target	Forward sequence	Reverse sequence
mouse *18S*	5′‐TCAACACGGGAAACCTCAC‐3′	5′‐CGCTCCACCAACTAAGAAC‐3′
mouse *Mtnr1a*	5′‐GCGTCATCGGCTCCATATTCAACA‐3′	5′‐CGTACTTGAGACTGTGGCAGATGTAG‐3′
mouse *Rubicon*	5′‐GAAGACGACTGTGGAAGGTTTG‐3′	5′‐AGCCCATGATACAGGATGTTCT‐3′
mouse *Uvrag*	5′‐ACATCGCTGCTCGGAACATT‐3′	5′‐CTCCACGTCGGATTCAAGGAA‐3′
mouse *Nox2*	5′‐TCAAGACCATTGCAAGTGAACAC‐3′	5′‐TCAGGGCCACACAGGAAAA‐3′

### 2.11 Immunofluorescence Analysis

Cells were cultured on glass coverslips and fixed in 4% PFA for 15 min before detergent extraction with 0.25% Triton X‐100 (93443, Sigma, California, USA) for 10 min at room temperature. Coverslips were saturated with 5% bovine serum albumin (V900933, Sigma, California, USA) in PBS for 30 min at room temperature and processed for immunofluorescence with the following primary antibodies: Iba1 (1:1000; AB_2224402; Abcam, Cambridge, UK), Map2 (1:1000; 4542S, CST, MA, USA), ps129‐α‐Syn (1:500; 015‐25191; Wako, Japan), Rubicon (1:100; PA5‐38017, Sigma, California, USA), followed by Donkey anti‐Goat IgG, Alexa Fluor 647 (1:500; A32849TR, Invitrogen, CA, USA), Donkey anti‐Mouse IgG, Alexa Fluor 594; (1:500; A21429, Invitrogen, CA, USA), Donkey anti‐Rabbit IgG, Alexa Fluor 488 (1:500; A11056, Invitrogen, CA, USA). Images were taken with the Zeiss LSM 900 confocal microscope.

### 2.12 Statistical Analysis

Throughout the paper, all analyses were performed using GraphPad Prism 8 (GraphPad Software, La Jolla, CA, USA). Before conducting statistical analyses, normality tests were performed on all data. The Shapiro–Wilk test was used to assess the normality of the data distribution. For data that exhibited a normal distribution, parametric tests such as the *t*‐test or analysis of variance (ANOVA) were applied. For data that did not exhibit a normal distribution, non‐parametric tests such as the Mann–Whitney *U* test or the Kruskal–Wallis test were used. All values are displayed as mean ± SEM. *p* < 0.05 was considered significant (*p < 0.05, **p < 0.01, ***p < 0.001; ns, not significant).

## 3 Results

### 3.1 MT1 Deletion Impaired the Phagocytic Function of Microglia

**FIGURE 1:**
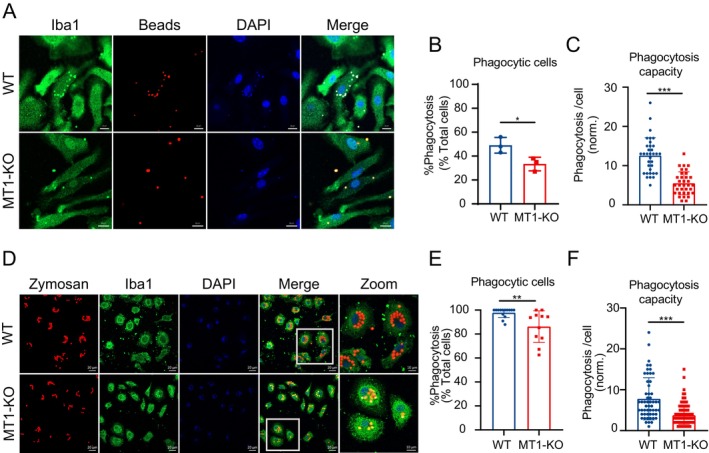
MT1 deletion impaired the phagocytic function of microglia. (A–C) Representative immunofluorescence images (A) and quantification (B, C) of fluorescence latex beads engulfed by WT and MT1‐KO primary microglial cells. Scale bar: 20.0 μm. Phagocytic cells (B) = number of cells phagocytosing latex beads/total cell count. Phagocytic capacity (C) = number of latex beads phagocytosed per single cell (*n* > 100 cells per group for analysis. *T*‐test, Mean ± SEM. *p < 0.05, ***p < 0.001 vs. WT group). (D–F) Representative immunofluorescence images (D) of fluorescence zymosan particles engulfed by WT and MT1‐KO primary microglial cells and the quantification of phagocytic cells (E) and phagocytic capacity (F). Scale bar: 20.0 μm (*n* > 100 cells per group for analysis. *T*‐test, Mean ± SEM. **p < 0.01, ***p < 0.001 vs. WT group).

To assess the impact of MT1 on the phagocytic function of microglial cells, we isolated primary microglial cells from both WT and MT1‐KO mice (Figure S1A,B). Firstly, we examined the phagocytic functionality of primary microglial cells within two distinct groups, employing fluorescent latex beads. The results revealed a significant decrease in both the percentage of cells engulfing latex beads and the number of beads phagocytosed per cell in the MT1‐KO group (Figure 1A–C), with the number of engulfed latex beads even decreasing by more than half (Figure 1C). Considering the non‐degradable and non‐biological characteristics of latex beads, we expanded our analysis to evaluate cell phagocytosis using fluorescent zymosan particles. In line with the observations made with latex beads, the MT1‐KO group displayed a pronounced impairment in phagocytic cells and a diminished phagocytic capacity (Figure 1D–F). However, MT1‐KO primary microglial cells, although compromised in phagocytic function, still demonstrated the ability to engulf zymosan particles in the majority of cells (approximately 80%) (Figure 1E). This may be attributed to the fact that primary microglial cells more closely resemble the state of in vivo microglial cells and are more sensitive to biological stimuli.

Meanwhile, we designed a small interfering RNA (siRNA) to target the *Mtnr1a* gene to knock down MT1 in BV2 cells and assessed the phagocytic capacity of BV2 cells, utilizing fluorescent latex beads and fluorescent zymosan particles. Encouragingly, MT1 knockdown in BV2 cells also resulted in a significant decrease in both the percentage of cells engulfing latex beads and the number of beads engulfed per cell (Figure S2A–C). Consistent with the findings with latex beads, the si‐*Mtnr1a* group exhibited significantly impaired phagocytic cell count and phagocytic capacity for fluorescent zymosan particles (Figure S2D–F).

Together, these data collectively indicate that MT1 plays a pivotal role in the phagocytic process of microglial cells, and its deletion can impair the phagocytic function of these cells.

### 3.2 MT1 Deficiency Impaired the Phagocytosis and Clearance of α‐Syn in Microglia

**FIGURE 2:**
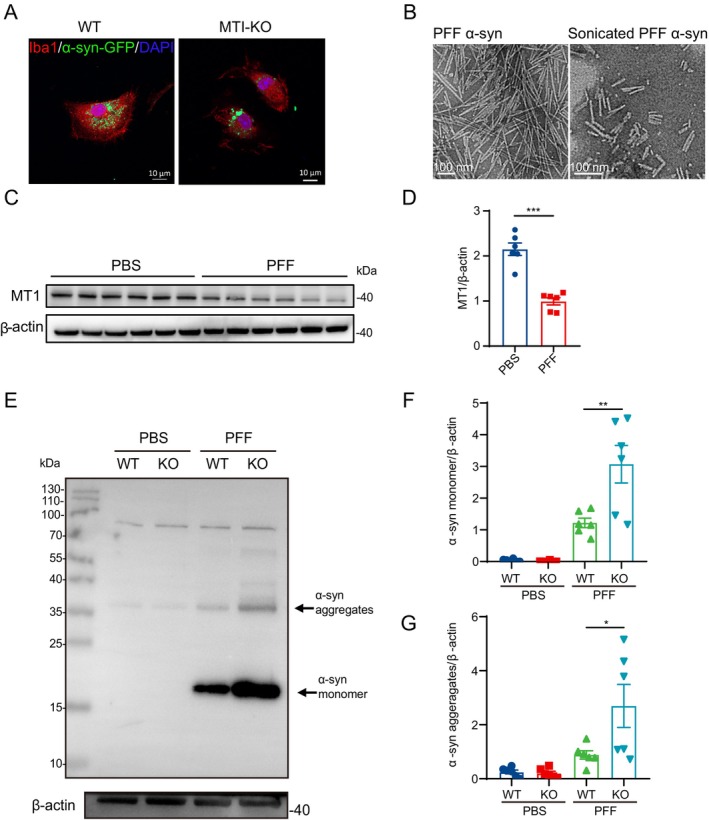
MT1 deficiency impaired the phagocytosis and clearance of α‐Syn in microglia. (A) Immunofluorescence analyses reveal the phagocytic uptake of α‐Syn–GFP monomers by primary microglial cells derived from WT and MT1‐KO mice. Scale bar: 10.0 μm. (B) Transmission electron microscopy images depict the fibrous structure of both pre‐ and post‐sonication of α‐Syn PFF. Scale bar: 100.0 nm. (C, D) Western blot (C) and group data (D) for MT1 levels in primary microglial cells treated by α‐Syn PFF for 24 hours (*n* = 6. *T*‐test, Mean ± SEM. ***p < 0.001 vs. PBS group). (E–G) Western blot (E) and the quantification of α‐Syn monomer (F) and α‐Syn aggregates (G) in primary microglial cells from both WT and KO groups after a 6‐h exposure to PBS or α‐Syn PFF (10 μg/mL), followed by a medium change and a subsequent 24‐h incubation (*n* = 6. One‐way ANOVA followed by Tukey's post hoc analysis. Mean ± SEM. *p < 0.05, **p < 0.01 vs. as indicated).

Given MT1's impact on microglial cell phagocytosis, we further explore its role in clearing pathological substances like α‐Syn within these cells. We treated primary microglial cells from both WT and MT1‐KO mice with α‐Syn‐GFP, assessing their monomeric α‐Syn engulfment ability 6 h post‐treatment. Our results indicated reduced α‐Syn‐GFP phagocytosis by MT1‐KO microglial cells compared to the WT group (Figure 2A). To better mimic the pathological α‐Syn aggregation observed in PD patients, we generated well‐characterized recombinant human α‐Syn and subjected it to sonication, yielding α‐Syn PFF with uniform dimensions ranging from 50 to 100 nm in length (Figure 2B). Notably, α‐Syn PFF treatment resulted in significant suppression of MT1 expression I primary microglial cells (Figure 2C,D) and BV2 cells (Figure S3A,B), while MT2 expression levels remained unaffected (Figure S3C,D) in primary microglial cells, which underscores the potentially pivotal role of MT1 of microglial cell in α‐Syn pathology.

Subsequently, primary microglial cells from both WT and MT1‐KO mice were exposed to α‐Syn PFF for 6 h and then cultured for an additional 24 h to assess their clearance of α‐Syn. The results revealed a diminished ability of MT1‐KO microglial cells to clear α‐Syn, leading to an exacerbated aggregation of α‐Syn monomers and aggregates in cells (Figure 2E–G). Consequently, the attenuated phagocytic activity of α‐Syn in microglial cells following MT1 knockout resulted in a diminished clearance of α‐Syn, ultimately contributing to increased pathological intracellular aggregation of α‐Syn.

### 3.3 MT1 Regulated LC3‐Associated Phagocytosis in Microglial Cells

Since the participation of MT1 in the phagocytic process of microglial cells and its regulatory role in the clearance and degradation of α‐Syn, exploring the potential underlying mechanisms becomes imperative. Recent research has unveiled a distinctive form of phagocytosis termed LAP. Therefore, we proceeded to investigate whether there is a correlation between MT1 and LAP.

**FIGURE 3:**
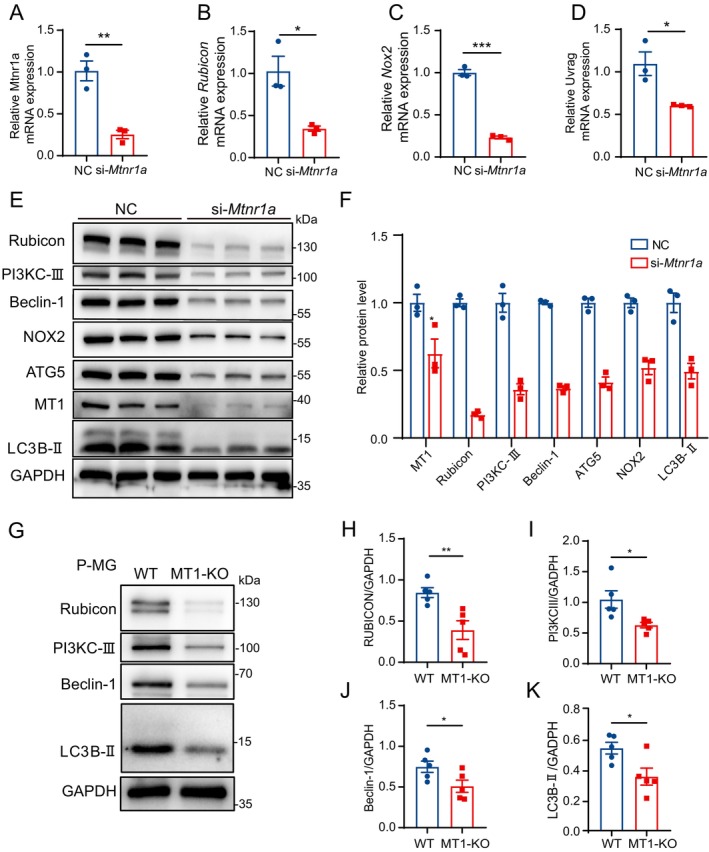
MT1 regulated LC3‐associated phagocytosis in microglial cells. (A–D) The mRNA expression levels of *Mtnr1a* (A), *Rubicon* (B), *Nox2* (C), and *Uvrag* (D) in BV2 cells 36 h post‐treatment with either negative control (NC) or si‐*Mtnr1a* small interfering RNA. 18S serving as internal controls (*n* = 3. *T*‐test, Mean ± SEM. *p < 0.05, **p < 0.01, ***p < 0.001 vs. NC group). (E, F) Western blot image (E) of the protein expression level of MT1, Rubicon, PI3KC‐III, Beclin‐1, NOX2, ATG5, LC3B‐II in NC, and si‐*Mtnr1a* groups and their relative protein quantification (F). GAPDH serves as internal control (*n* = 3. *T*‐test, Mean ± SEM. *p < 0.05, **p < 0.01, ***p < 0.001 vs. NC group). (G–K) Western blot image (G) of the protein expression level of Rubicon, PI3KC‐III, Beclin‐1, LC3B‐II in WT, and MT1‐KO microglia and their relative protein quantification (H–K). GAPDH serves as internal control (*n* = 3. *T*‐test, Mean ± SEM. *p < 0.05, **p < 0.01 vs. WT group).

Upon silencing *Mtnr1a* in BV2 cells, we scrutinized the mRNA expression levels of LAP‐related genes. Notably, the downregulation of *Mtnr1a* (Figure 3A) corresponded with a parallel decrease in the mRNA expression levels of *Rubicon*, *Uvrag*, and *Nox2* (Figure 3B–D). Considering the influence of gene expression on protein translation, we employed Western blotting to assess alterations at the protein level. In harmony with the genetic‐level findings, MT1 knockdown triggered alterations in LAP‐related molecules (Figure 3E). These changes included a decrease in the expression of upstream molecules crucial for phagosome formation and the formation of the PI3KC‐III complex within the LAP pathway, such as Rubicon, PI3KC‐III, UVRAG, and Beclin‐1. Furthermore, key molecules governing ROS generation, notably NOX2, exhibited decreased. Simultaneously, the expression levels of LC3‐II and ATG5, integral to LC3‐II generation, were also diminished (Figure 3F). This suggests a potential association between the observed decline in phagocytic ability in BV2 cells following MT1 knockdown and LAP impairment.

Additionally, we evaluated the expression levels of pertinent proteins in primary microglial cells obtained from WT and MT1‐KO mice (Figure 3G). We also observed a decrease in the expression levels of RUBICON, PI3KC‐III, Beclin‐1, and LC3 following MT1 deletion (Figure 3H–K). In summary, these findings suggest that MT1 potentially participates in the phagocytic process of microglial cells by modulating LAP.

### 3.4 MT1‐Rubicon Interaction: Enhancing LAP Through MT1 Overexpression or Activation

**FIGURE 4 |:**
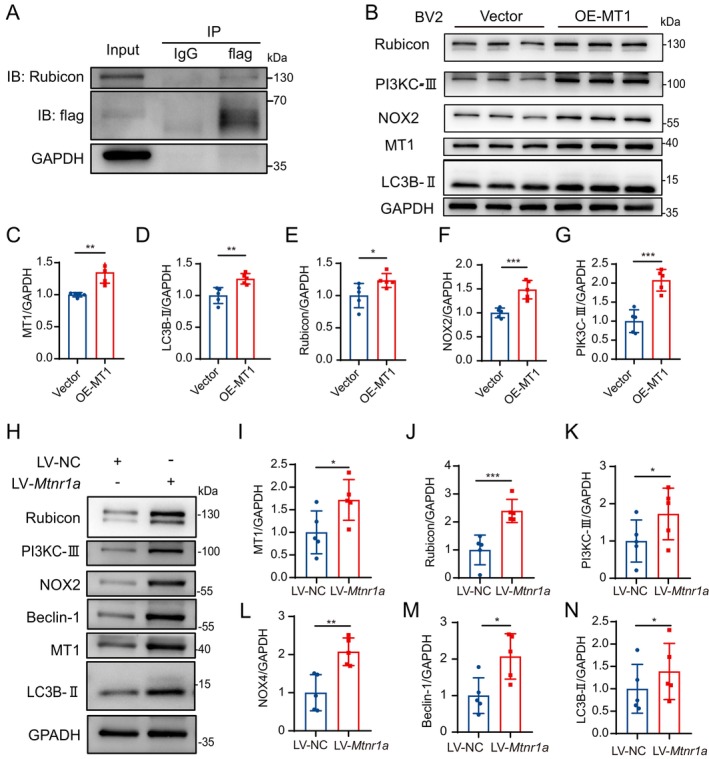
MT1–Rubicon interaction: Enhancing LAP through MT1 overexpression or activation. (A) Co‐IP validation of MT1 and Rubicon interaction in MT1‐overexpressing BV2 cell line. (B–G) Western blot image (B) of the protein levels of MT1 and LAP‐related protein in BV2 cells transfecting Vector or MT1 overexpression plasmid for 36 h. The relative protein quantification of MT1 (C), LC3B‐II (D), Rubicon (E), NOX2 (F), and PI3KC‐III (G). GAPDH was used as internal controls (*n* = 3. *T*‐test, Mean ± SEM. *p < 0.05, **p < 0.01, ***p < 0.001 vs. NC group). (H–N) Western blot image (H) of the protein levels of MT1 and LAP‐related protein in primary microglial cells transfecting NC or MT1 overexpression lentiviral for 120 h. The relative protein quantification of MT1 (I), Rubicon (J), PI3KC‐III (K), NOX2 (L), Beclin‐1 (M), and LC3B‐II1 (N). GAPDH was used as an internal control (*n* = 4. *T*‐test, Mean ± SEM. *p < 0.05, **p < 0.01, ***p < 0.001 vs. NC group).

To delve deeper into the involvement of MT1 in the phagocytic process of microglial cells, we engineered a lentivirus overexpressing MT1 and established a stable MT1‐overexpressing BV2 cell line. Following this, we performed co‐immunoprecipitation (co‐IP) analysis on this BV2 cell line to validate the interaction between MT1 and Rubicon (given that Rubicon is a key signaling molecule in the LAP process) (Figure 4A). Moreover, we overexpressed MT1 by transfecting a human Mtnr1a plasmid into BV2 cells, resulting in elevated MT1 protein expression and concurrent increases in LAP‐associated proteins, including Rubicon, PI3KC‐III, NOX2, and LC3B‐Ⅱ (Figure 4B–G). Meanwhile, we overexpressed the *Mtnr1a* lentivirus in primary microglial cells, and the expression levels of LAP‐associated proteins such as Rubicon, PI3KC‐Ⅲ, NOX2, BECLIN1, and LC3‐II increased concomitantly with the overexpression of MT1. This finding further validates our results obtained from MT1‐KO primary microglial cells (Figure 4H–N). Additionally, the MT1 receptor agonist, Ramelteon, was applied to BV2 cells to observe the effects of MT1 activation on LAP. The results revealed that while there were no significant changes in the protein expression of MT1 itself, its functional activation led to an increase in Rubicon and LC3‐II (Figure S4A,B). Following stimulation with Ramelteon in primary microglial cells, there was a notable enhancement in Rubicon expression, as evidenced by immunofluorescence demonstrating an increased co‐localization with the microglial marker Iba1 (Figure S4C). In summary of the aforementioned results, it is conceivable that MT1 may, through its interaction with Rubicon, further initiate the occurrence of the LAP process.

### 3.5 Microglial MT1 Regulated the Clearance and Degradation of Pathological α‐Synuclein in Neurons

**FIGURE 5:**
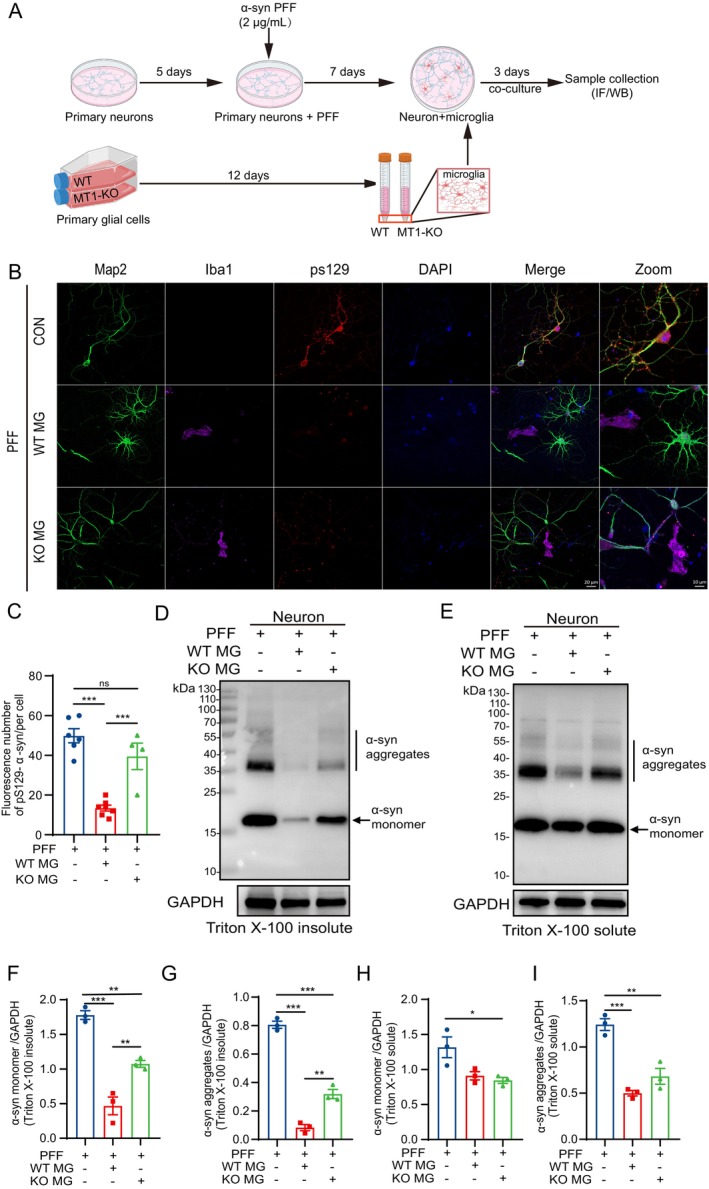
Microglial MT1 regulated the clearance and degradation of pathological α‐Syn in neurons. (A) Schematic depiction of the construction of the neuron–microglia co‐culture model. (B, C) Immunofluorescent image (B) of pS129 α‐Syn in co‐culture models of WT or MT1‐KO primary microglial cells with α‐Syn PFF‐induced pathological neurons, along with the corresponding statistical analysis of the number of pS129 α‐Syn (C) (MG = primary microglia, *n* > 10 cells per group for analysis. One‐way ANOVA followed by Tukey's post hoc analysis. Mean ± SEM. ***p < 0.001 vs. as indicated; ns, not significant). (D–I) Western blot images of the expression of α‐Syn in Triton‐insoluble (D) and Triton‐soluble (E) protein fractions of neuron–microglia co‐cultures. Quantitative analysis of Triton‐insoluble α‐Syn monomer (F) and aggregates (G), as well as Triton‐soluble α‐Syn monomer (H) and aggregates (I). GAPDH was used as control. (MG = primary microglia, *n* = 3. One‐way ANOVA followed by Tukey's post hoc analysis. Mean ± SEM. *p < 0.05, **p < 0.01, ***p < 0.001 vs. as indicated; ns, not significant).

In our preceding results, we elucidated the involvement of MT1 in the phagocytosis and degradation of α‐Syn by microglial cells. However, in the progression of PD, α‐Syn primarily aggregates within neurons initially and is subsequently released into the extracellular environment, where microglial cells recognize and engage in processing [51]. To emulate this scenario, we established a primary neuron–microglia co‐culture model (Figure 5A). Alpha‐synuclein can manifest in various aggregate forms, with phosphorylation at serine 129 (pS129‐α‐Syn) serving as a hallmark modification in pathological α‐Syn [52]. Thus, IF staining for the neuronal marker Map2, microglial marker Iba1, and pS129‐α‐Syn was performed in a co‐culture model (Figure 5B). Neurons induced by α‐Syn PFF exhibited a notable accumulation of pS129‐α‐Syn. Co‐culturing neurons with WT microglial cells significantly attenuated pS129‐α‐Syn aggregation, whereas co‐culturing with MT1‐KO microglial cells resulted in a heightened aggregation of pS129‐α‐Syn (Figure 5C). Furthermore, soluble and insoluble α‐Syn fractions were isolated from the neuron–microglia co‐culture models. The MT1‐KO microglial cell co‐culture group demonstrated heightened expression levels of insoluble α‐Syn monomers and aggregates, particularly in the aggregated form, compared to the WT group (Figure 5D,F,G). Simultaneously, a noticeable upward trend was evident in soluble α‐Syn aggregates (Figure 5E,H,I). These findings suggest that microglial cells possess the capability to eliminate aberrantly aggregated α‐Syn in pathological neurons. The deletion of MT1 impedes this capacity, underscoring the involvement of microglial cell MT1 in the clearance and degradation process of pathological α‐Syn aggregates induced by α‐Syn PFF.

## 4 Discussion

In our study, we have illuminated the role of MT1 in the microglial phagocytic process and its influence on the progression of PD. We proposed that the MT1 could improve microglial phagocytic function through ameliorating Rubicon‐dependent LAP signaling, thereby participating in the aggregation and clearance of pathological α‐Syn induced by α‐Syn PFF in neurons, which might be the potential mechanisms to interpret the neuroprotective properties of MT1. The current study provides novel perspectives into how microglial MT1 alters both microglial function and the underlying mechanisms in α‐Syn pathology.

PD is a neurodegenerative disease intricately linked with the aging process, whose prominent pathological feature is the formation of Lewy bodies in neurons, primarily composed of abnormally aggregated α‐Syn. Existing research indicates that mutations or misfolding of the SNCA gene, responsible for encoding α‐Syn, can instigate the aggregation of α‐Syn proteins into oligomers, fibrils, or Lewy bodies. These aggregates often exert cytotoxic effects, triggering a cascade of pathological responses, including oxidative stress, inflammation, and autophagic dysfunction, ultimately culminating in cell death. While previous studies predominantly focused on elucidating the neurotoxicity and damage caused by the pathological aggregation of α‐Syn to dopaminergic neurons, recent studies have unveiled a novel perspective. It has been observed that α‐Syn accumulating within dopaminergic neurons can be released extracellularly through processes like exocytosis, subsequently spreading across diverse cells within the neural network. This phenomenon generates more extensive physiological and pathological effects [53]. In this context, the activation and phagocytic activity of microglia emerge as pivotal factors in the clearance and maintenance of brain homeostasis, particularly in the degradation of α‐Syn. Hence, the identification of novel targets associated with phagocytosis holds paramount significance in furthering our comprehension of microglial function in neurodegenerative diseases.

MT1, being among the affinity receptors for melatonin, experiences a certain degree of downregulation in the brain as age advances [54]. They hold a pivotal role in neurological disorders. Research suggests that the deficiency of MT1 increases the risk of AD and exacerbates the toxic damage of protein aggregates to neurons in Huntington's disease [55, 56]. An earlier study has shown higher MT1 expression in microglial cells compared to astrocytes and neurons [42]. This study further identified a decrease in MT1 expression levels in microglial cells after treatment with α‐Syn PFF, while MT2 showed no significant change. Consequently, we propose that the deficiency or dysfunction of MT1 in microglial cells may contribute to the pathological progression of α‐synuclein. Our experiments focused primarily on in vitro cellular and molecular studies, utilizing immortalized BV2 cell lines, primary microglial cells, and primary neurons as models to address the scientific questions. Crucially, the primary microglial cells were derived from C57BL/6 mice with the *Mtnr1a* gene knocked out, which provided a more robust validation of MT1 functionality. Our observation is that MT1 can regulate the phagocytic function of microglial cells. Specifically, the knockout of MT1 results in a downregulation of the expression levels of molecules involved in LAP, while overexpressing or activating MT1 exerts a positive regulatory effect. LAP involves three stages: formation of the phagosome, formation of the PI3KC‐3 complex, and connection of LC3‐II with the phagosome. Among these stages, Rubicon stands out as LAP‐specific, playing a vital role in both phagosome formation and ROS release. Recognized as a LAP marker, it typically exerts its influence during the initial stages of LAP [28, 57, 58]. Our findings indicate an MT1–Rubicon interaction, suggesting that MT1 regulates the series of LAP reactions through this interaction. Manipulating MT1 induces changes in associated molecules, supporting its role as a key upstream molecule in the LAP of microglial cells.

In recent years, there has been a heightened focus on elucidating the intricate interplay between microglial cells and neurons. Most studies have primarily focused on detecting the stimulating effects of components such as cytokines released by microglial cells on neurons. While this approach partially encapsulates the functionality of microglial cells, it often overlooks the fundamental premise of cellular coexistence. To overcome this limitation, our study implemented a specialized neuron–microglia co‐culture model, facilitating reciprocal contact and cohabitation. This model authentically reproduces the in vivo milieu, enabling a more nuanced exploration of the microglial cell‐neuron relationship. Within this model, we discerned that the genetic knockout of microglial cell MT1 exerted a discernible influence on the pathological aggregation of α‐Syn in neurons induced by α‐Syn PFF, particularly evident in the formation of insoluble α‐Syn aggregates. This disparity is likely attributed to alterations in LAP mediated by MT1.

Although we have observed changes in molecules related to the initiation of LAP and phagosome formation, the processes of lysosome‐phagosome fusion and pathological protein degradation remain unexplored issues in this study. Besides, our study currently lacks in vivo evidence; however, related research from our group indicates that the knockout of MT1 exacerbates the loss of dopaminergic neurons and motor dysfunction in a PFF‐induced pathological mouse model of PD, which suggests that MT1 plays a critical role in the neurodegenerative processes associated with PD [59]. Moreover, examining whether the conditional knockout of microglial cell MT1 in mice intensifies α‐Syn aggregation, resulting in more pronounced motor and non‐motor symptoms of PD, will contribute to a more comprehensive understanding of the role of MT1 in microglial cells. As we all know, the ultimate goal of scientific research is to translate basic pathological mechanism studies into clinical applications and treatments. Therefore, research involving human cells and tissues holds greater significance. By using induced pluripotent stem cells (iPSCs) to differentiate into microglia and specifically regulate the expression of MT1, we can better mimic the role of MT1 in PD and other human disorders. Currently, melatonin receptor agonists applied to clinical patients include ramelteon and agomelatine, mainly used for the treatment of sleep disorders and depression. Some studies suggest that selective MT1 agonists may be potential candidates for regulating circadian rhythms and sleep [60]. Our study indicates that MT1 also plays a role in the phagocytosis of microglial cells and the clearance of pathological proteins. Therefore, the development of MT1‐related targeted compounds and drugs may assist in precision pathological treatment for PD patients.

In conclusion, our study has elucidated the role of MT1 in microglial phagocytosis, indicating that MT1 protein regulates LAP and is involved in the clearance and degradation of pathological α‐Syn, thereby alleviating the aggregation of α‐Syn in neurons. Finally, with further research and development, MT1 is expected to become a new target for the treatment of PD and related α‐synucleinopathies.

## Author Contributions

C.‐F.L. and F.W. designed research; X.‐Y.Y., B.‐E.C., J.‐Y.L., X.‐Y.C., J.‐R.Z., and Q.‐K.L. performed experiments; X.‐Y.Y. and B.‐E.C. analyzed data and wrote the manuscript; Q.‐H.M., C.‐J.M., F.W., and C.‐F.L. revised the manuscript.

## Conflicts of Interest

The authors declare no conflicts of interest.

## Supporting information


**Figures S1** **–S4**

## Data Availability

The data that support the findings of this study are available from the corresponding author upon reasonable request.
