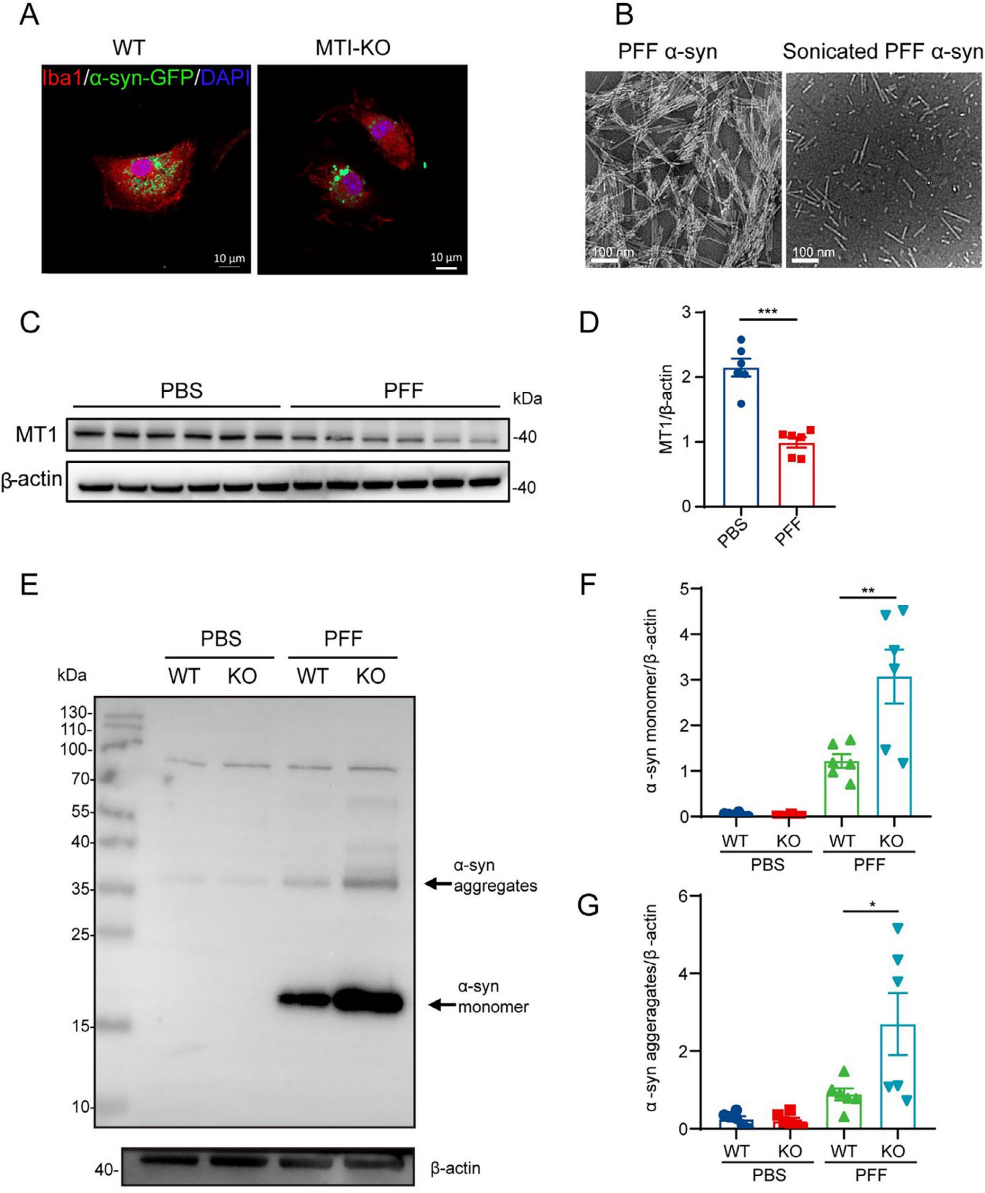# Correction to “Microglial Melatonin Receptor 1 Degrades Pathological Alpha‐Synuclein Through Activating LC3‐Associated Phagocytosis In Vitro”

**DOI:** 10.1111/cns.70219

**Published:** 2025-01-13

Yao XY, Cao BE, Liu JY, Lv QK, Zhang JR, Cheng XY, Mao CJ, Ma QH, Wang F, Liu CF. “Microglial Melatonin Receptor 1 Degrades Pathological Alpha‐Synuclein Through Activating LC3‐Associated Phagocytosis In Vitro”. *CNS Neurosci Ther*. 2024;30(10):e70088. doi: 10.1111/cns.70088.

The authors would like to correct Figure 2B, as errors were made during its preparation for publication. We declare that these corrections do not alter the results or conclusions of this paper. The corrected version of Figure 2, including Figure 2B, is provided here.

We sincerely apologize for the article's error and any inconvenience caused.