# Supplementary material for: Microglial Melatonin Receptor 1 Degrades Pathological Alpha‐Synuclein Through Activating LC3‐Associated Phagocytosis In Vitro
Source: CNS Neurosci Ther. 2024 Oct 23;30(10):e70088. doi: 10.1111/cns.70088 (PMC11499215; doi:10.1111/cns.70088)
Supplement: Supplementary file 1 — Figures S1 –S4 [file CNS-30-e70088-s001.zip › Supplemental figure-1020.docx]

**Supplemental figure legends**

**Figure S1.** Mouse gene identification and microglial cell purity.

(A) Gene identification image for *Mtnr1a* knockout mice: *Mtnr1a*^+/+^: wild-type (WT) mice; *Mtnr1a*^+/-^: heterozygous mice. *Mtnr1a*^-/-^: *Mtnr1a* knock-out (MT1-KO) mice. (B) Immunofluorescence staining for purity identification of primary microglial cells: Iba1 is a marker to assess the purity of primary microglial cells—scale bar: 100 μm.

**Figure S2.** MT1 knockdown reduces phagocytic function in BV2 cells.

BV2 cells were treated with Negative control (NC) and si-*Mtnr1a* small interfering RNA for 36 hours.

(A-C) Representative immunofluorescence images (A) and quantification (B-C) of fluorescence latex beads engulfed by BV2 cells in NC and si-*Mtnr1* group. Scale bar: 20.0 μm. Phagocytic cells (B) = number of cells phagocytosing latex beads / total cell count. Phagocytic capacity (C) = number of latex beads phagocytosed per single cell. (n > 100 cells per group for analysis, T-test, Mean ± SEM. **p < 0.05*, ****p < 0.001* versus NC group).

(D-F) Representative immunofluorescence images (D) depict fluorescence zymosan particles engulfed by BV2 cells in both the NC and si-Mtnr1 groups, accompanied by quantifications of phagocytic cells (E) and phagocytic capacity (F). Scale bar: 20.0 μm. (n > 100 cells per group for analysis, T-test, Mean ± SEM. ****p < 0.001* versus NC group).

**Figure S3.** Decreased MT1 expression of BV2 cells and unaltered MT2 expression in primary microglial cells after PFF treatment.

(A-B) Western blot (A) and group data (B) for MT1 levels in BV2 cells treated by α-Syn PFF for 24 hours (n =3. T-test, Mean ± SEM. ***p < 0.01* versus PBS group t).

(C-D) Western blot (C) and group data (D) for MT2 levels in primary microglial cells treated by α-Syn PFF for 24 hours (n =3. T-test, Mean ± SEM. ns, not significant).

**Figure S4.** The melatonin receptor agonist stimulates the expression of LAP-related proteins.

(A-B) Western blot image (A) and quantification of MT1, Rubicon, and LC3B-Ⅱ (B) in BV2 cells treated with DMSO or Ramelteon (100 μM) for 24 hours (n =4. T-test, Mean ± SEM. **p< 0.05* versus DMSO group; ns, not significant).

(C) Immunofluorescence image showed the co-localization of Rubicon and Iba1 following a 24-hour treatment of primary microglial cells with Ramelteon (100 μM). Scale bar: 20.0 μm.


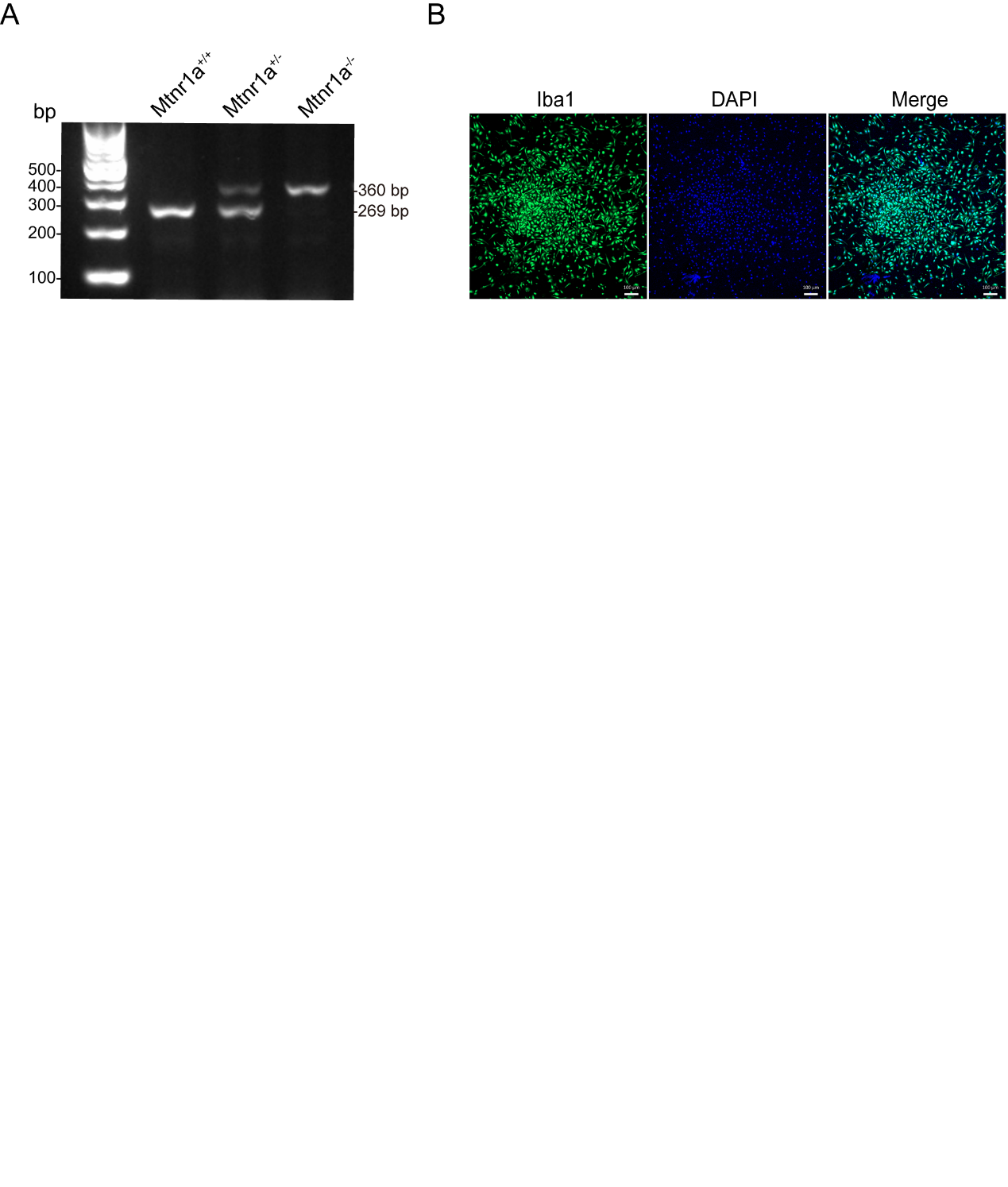


**Figure S1**


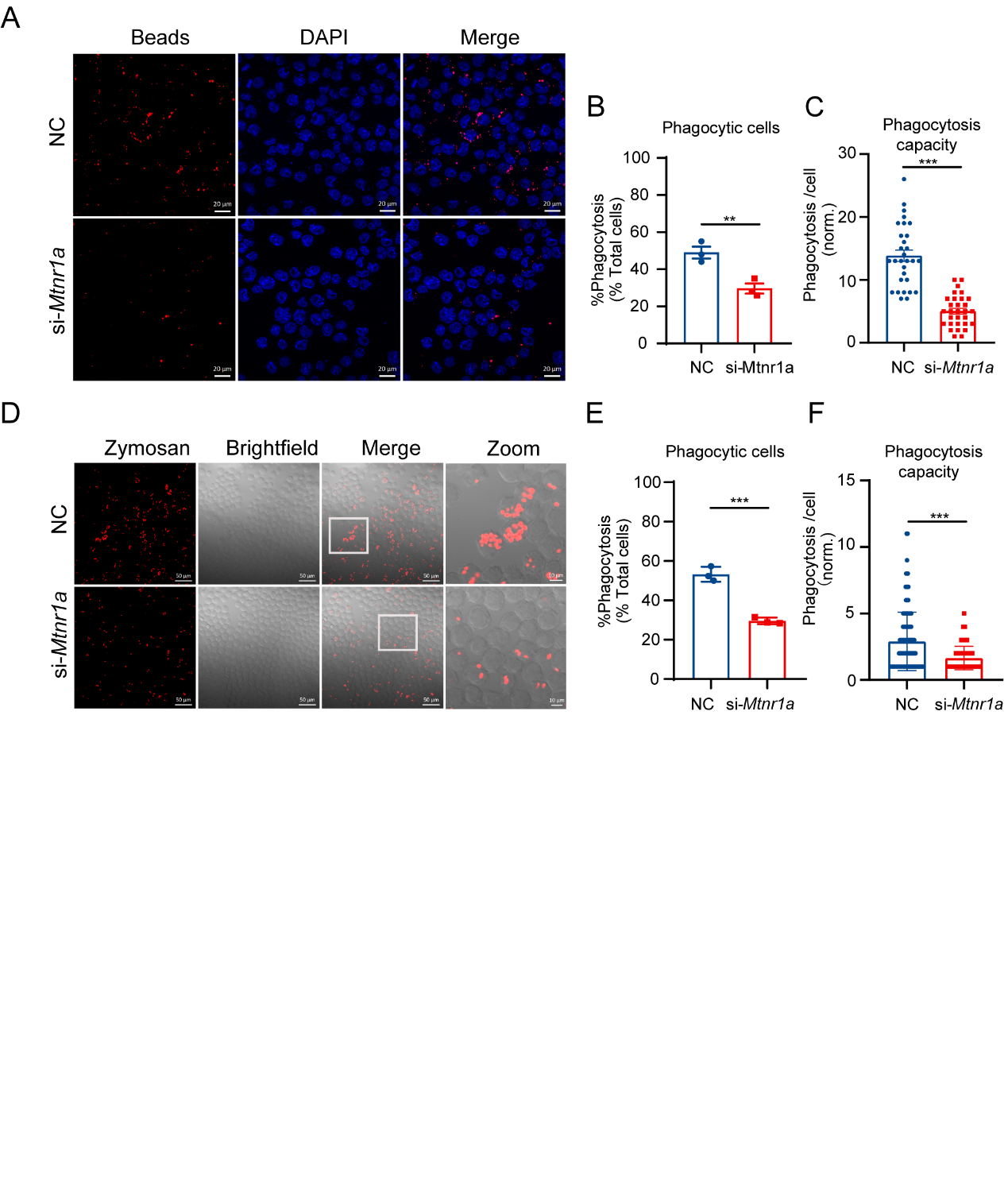


**Figure S2**


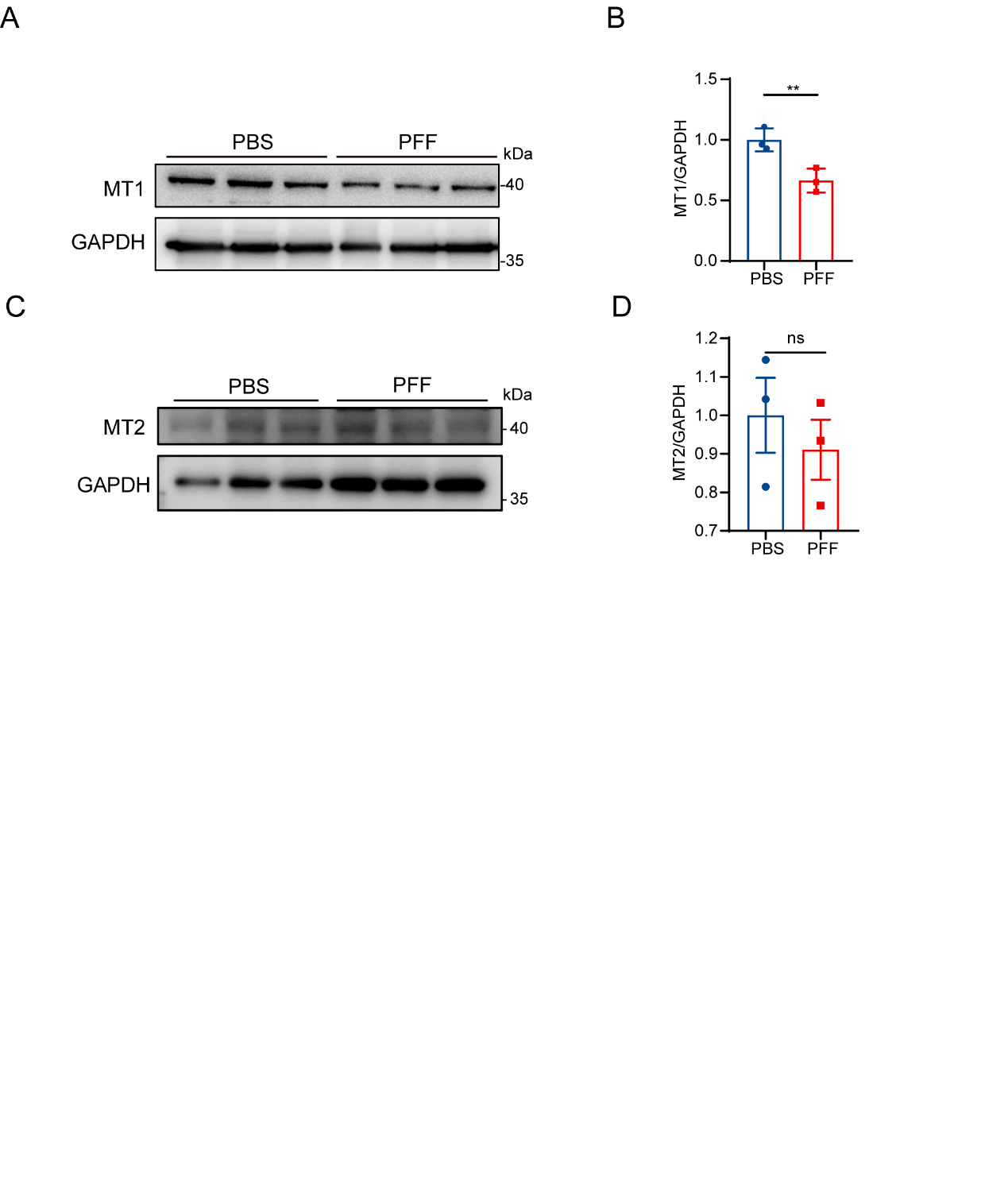


**Figure S3**

**
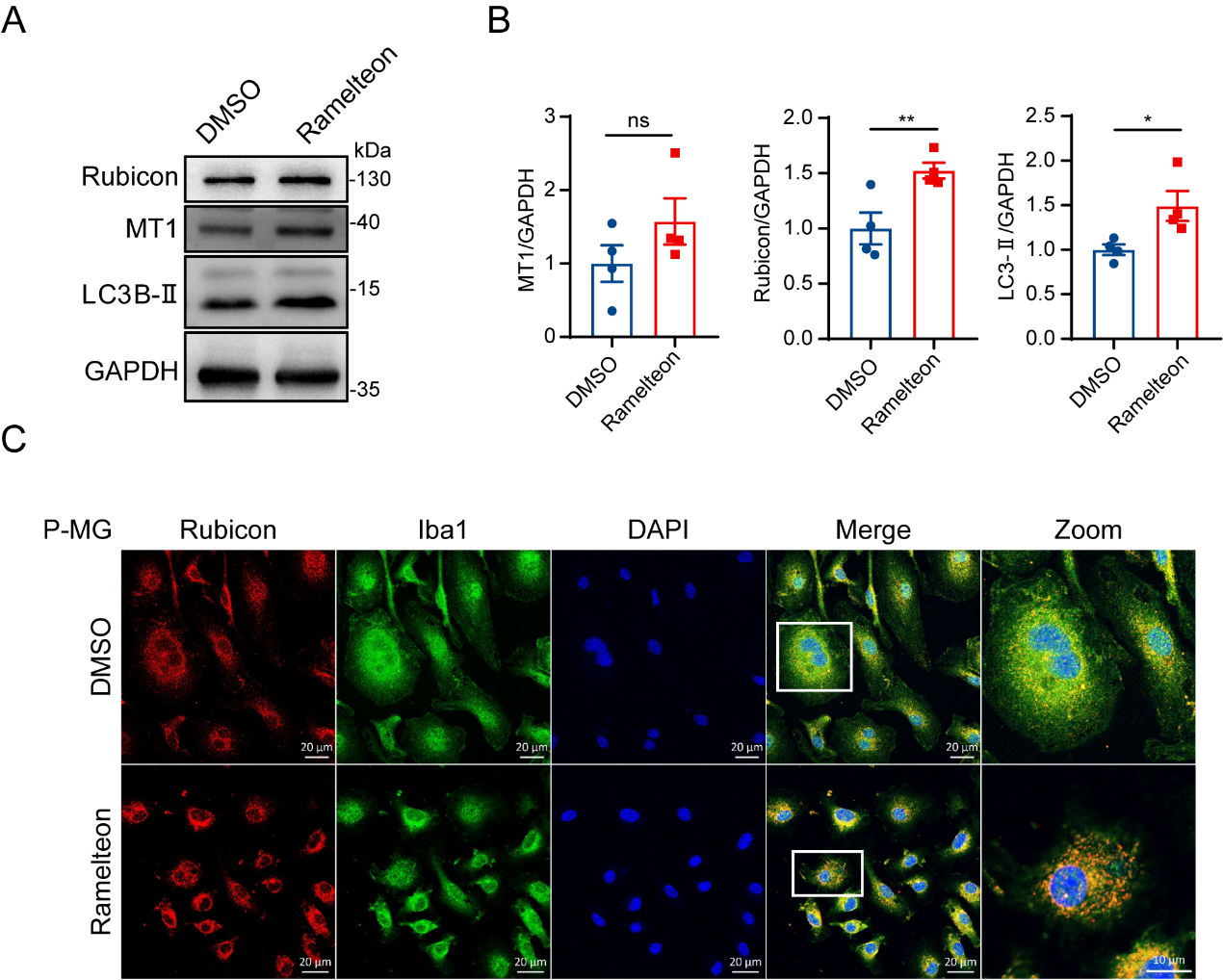
Figure S4**
